# Fabrication of Functional Plastic Parts Using Nanostructured Steel Mold Inserts

**DOI:** 10.3390/mi8060179

**Published:** 2017-06-06

**Authors:** Nicolas Blondiaux, Raphaël Pugin, Gaëlle Andreatta, Lionel Tenchine, Stéphane Dessors, Pierre-François Chauvy, Matthieu Diserens, Philippe Vuillermoz

**Affiliations:** 1 Centre Suisse d’électronique et de Microtechnique CSEM, 1 rue Jaquet-Droz, CH-2002 Neuchatel, Switzerland; rpu@csem.ch (R.P.); gaa@csem.ch (G.A.); 2 Centre Technique Industriel de la Plasturgie et des Composites-IPC, 2 rue Pierre et Marie Curie, 01100 Bellignat, France; Lionel.TENCHINE@ct-ipc.com (L.T.); Stephane.DESSORS@ct-ipc.com (S.D.); 3 Micropat SA, 30 Côtes-de-Montbenon, CH-1003 Lausanne, Switzerland; pf@micropat.ch (P.-F.C.); mat@micropat.ch (M.D.); 4 Vuillermoz SAS, 5 rue du Tomachon, 39200 Saint-Claude, France; pv-vuillermoz@orange.fr

**Keywords:** nanostructures, hot embossing, injection molding, polymer, 3D, steel, mold, wettability, immunoassay

## Abstract

We report on the fabrication of sub-micro and nanostructured steel mold inserts for the replication of nanostructured immunoassay biochips. Planar and microstructured stainless steel inserts were textured at the sub-micron and nanoscale by combining nanosphere lithography and electrochemical etching. This allowed the fabrication of structures with lateral dimensions of hundreds of nanometers and aspect ratios of up to 1:2. Nanostructured plastic parts were produced by means of hot embossing and injection molding. Surface nanostructuring was used to control wettability and increase the sensitivity of an immunoassay.

## 1. Introduction

There is a growing trend for the fabrication of smart products with novel functionalities or enhanced performances. One route to achieve this goal is an accurate control of surface properties. Surface chemistry, topography and a combination of both can be engineered and optimized for specific applications. Surfaces with controlled topographies have, for example, been manufactured to reduce the adhesion of bacteria or living adherent cells [1,2] and to control friction and adhesion between surfaces [3]. The effect of surface roughness on wettability is a widely studied field [4]. Mimicking the well-known lotus effect has been the focus of many studies for the fabrication of superhydrophobic surfaces with controlled wetting states, for example, Cassie–Baxter vs. the Wenzel state [5,6]. As reported by Martines et al., the processing of silicon-based materials using advanced lithographic techniques allows the design and fabrication of surfaces with a high degree of control over surface chemistry and topography [7]. Plastic parts with controlled wettability have also been produced using replication techniques such as hot embossing and injection molding, however, the production of highly liquid-repellent plastic surfaces without any surface treatment remains challenging [8]. Another field of application relevant to this study is the use of surface micro- and nanostructuring to enhance the performance of sensors for biomedical and point-of-care diagnostics [9]. Highly sensitive plasmonic [10,11] and surface enhanced Raman spectroscopy (SERS) sensors have been produced by optimizing surface structures on metallic layers, with enhancement factors of up to 108 compared to on flat surfaces [12]. Another way to improve the sensitivity of sensors is to increase the specific area of the sensing element. This has been shown by Ingham et al., who used anodized alumina to create highly porous sensors with surface areas two to three orders of magnitude greater than flat surfaces [13]. Kim et al. also reported a significant increase in the fluorescence intensity (four times greater compared to a flat surface) after fabricating quartz nanopillars on the surface of a DNA biosensor [14]. Such an effect was also demonstrated by Kuwabara et al. [15] on polystyrene immunoassay chips using a nanoimprinting process. By using a specific elongation process during nanoimprinting, high aspect ratio pillars were produced, giving rise to a 34-fold increase in the fluorescence intensity.

The examples mentioned above confirm the potential benefits of surface micro- and nanostructuring and show that various techniques have been used to produce specific structures in different materials. One of the objectives of this study was to produce nanostructured, functional parts using replication techniques such as hot embossing and injection molding. One of the key points, therefore, is the fabrication of nanostructured mold inserts. Although the process chain developed for the production of CDs and DVDs is well established for the fabrication of nanostructured nickel stampers, there is an increasing interest to produce steel mold inserts. The main motivations are the ease of integration in standard injection molds, the wide range of materials with better durability and the possibility of combining different techniques to process inserts at different length scales without being limited by the thickness of the electroformed stamper. Conventional techniques such as micromilling, laser ablation and wire electrical discharge machining (EDM) are well established for the microstructuring of steels [16,17]. These are cost effective and allow the fabrication of high aspect ratio structures on freeform shapes, but have a resolution limited to few micrometers. Alternatively, advanced lithography techniques have been developed for the microelectronic industry during the last few decades. UV, X-ray, interference and e-beam lithography, when combined with other microfabrication processes such as thin film deposition and etching, have paved the way for the fabrication of nanostructured surfaces and devices [18,19]. Emerging bottom-up approaches such as block copolymer and nanosphere lithography have also appeared with the common goal of fabricating smaller structures [20,21]. These techniques are state of the art with regard to resolution, but they are limited to planar substrates and have mainly been applied to silicon-based materials. When combined with electroforming, structured nickel stamps form the basis of the production of CDs and DVDs. Several studies report the use of photolithography to fabricate structured steel surfaces. Compared to standard microstructuring techniques such as milling or EDM, this involves the fabrication of an etch mask, followed by the etching of the substrate. In photochemical etching, standard photolithography is combined with chemical etching for the fabrication of a micropart or for surface microstructuring as shown by Hao et al., Masuzawa et al. and Mason et al. [22,23,24]. However, one drawback of chemical etching is the significant increase in the surface roughness of the etched surface, which results from the chemical etching solution used and the microstructure of the steel. This can be overcome by using an electrochemical etching technique, which leads to a mirror-polished surface in the etched areas. Landolt et al. made comprehensive reviews on the effect of the main parameters affecting electrochemical etching for titanium and stainless steel surfaces [25,26,27]. Usually limited to an isotropic profile, electrochemical etching has also been used in a sequential Bosch-like process to create higher aspect ratio structures, as reported by Shimizu et al. [28]. Finally, physical etching techniques have also been used for the fabrication of nanostructured steel surfaces. An alternative to wet etching techniques has been proposed by Al-Azawil et al., who combined photolithography and ion beam etching for the surface structuring of injection mold inserts. The very low selectivity of ion beam etching allowed homogeneous etching of the steel surface despite the heterogeneity of the material (Cr, Ni content, presence of MnS inclusions, steel microstructure) [29]. This technique was also used by Kurhihara et al. with a layer of nanoparticles as an etch mask [30]. Although the nanoparticle layers used as etch masks are not as well defined as etch masks made by photolithography, this approach allowed the processing of curved injection molding dies for the fabrication of optical lenses with improved antireflectivity.

When specifically considering the surface structuring of injection mold inserts, the process chain developed for CDs and DVDs remains state of the art for the injection molding of nanostructured plastic parts. However, several limitations remain concerning the ease of integration of the nickel inserts in the molds, its durability, its compatibility with other techniques to make hierarchical structures, the processing of non-planar surfaces and the presence of nickel which is banned for medical applications. An alternative is the direct processing of steel grades being used for mold manufacturing. This allows standard techniques such as milling, EDM and laser ablation to be used to control the overall shape and macro-/microstructures of the insert, and surface micro- and nanostructuring to be added to the standard mold manufacturing chain. This approach also gives greater flexibility regarding the materials of the inserts and the means of integration into the existing mold. Therefore, the overall objective of this study is to develop new processes to engineer the surface roughness of steel inserts compatible with injection molding and to apply them to the production of nanostructured plastic parts. The main challenge is to apply surface structures with typical lateral dimensions (a few hundred nanometers) onto the multilevel microfeatures of a stainless steel insert. In achieving this goal, most of the techniques mentioned above would face limitations. Although they have sufficiently high resolution, standard techniques such as photo-, e-beam or interference lithography would not be feasible due to the tridimensional shape of the part to treat. Standard microstructuring techniques, such as EDM or micromilling, are 3D compatible but do not have a sufficiently high resolution. The process flow used for the fabrication of CDs and DVDs would be one way to produce multilevel structures but a major objective of this study is to propose an alternative to nickel inserts for injection molding for the aforementioned reason.

In this study, a new process chain for the surface structuring of steel inserts has been developed. The technique, based on the combination of nanosphere lithography and electrochemical etching, has been used to fabricate structured stainless steel inserts with sub-micro-/nanofeatures. The choice of these techniques was mainly due to their compatibility with 3D parts. The final goal of the project was the production of a bio-diagnostic platform capable of performing immunoassays with increased sensitivity. The functional part was a microscope slide with an array of detection spots (micropillars) located at the bottom of a microchannel. The objective was to introduce micro-/nanostructures on top of the detection spots to control the functionalization of the spots during the immunoassay (via control of wetting) and to enhance the fluorescence signal. Nanostructured 2D surfaces were produced by hot embossing as references, and nanostructured bio-diagnostic platforms were produced by injection molding. The effect of surface structuring on wettability was characterized by means of water contact angle measurements and a model immunoassay was carried out to investigate the effect on sensitivity of the detection of the bio-diagnostic platform.

## 2. Materials and Methods

### 2.1. Steel Substrate Preparation

Stainless steel (316L) was used. The different steps of the process chain were optimized using steel discs (30 mm diameter, 3 mm thickness). The surfaces of the discs were mirror-polished before surface nanostructuring using SiC grit paper and an alumina suspension. The final mold insert used for the injection molding of the bio-diagnostic platform was fabricated using conventional micromilling processes. A 500 μm wide, 150 μm high ridge, corresponding to the microchannel on the plastic part, was first micromilled. An array of microholes (diameter: 300 μm, depth: 100 μm) was then fabricated on top of the ridge. The bottom of the fabricated microholes was then polished by through-mask electrochemical micromachining of the stainless steel [25]. The mirror-polished discs (also referred to as 2D substrates in the text) and microstructured steel inserts (also referred to as 3D substrates) were passivated using a nitric acid solution (20% *v*/*v* in water, 60 °C, 30 min) and thoroughly rinsed with deionized water.

### 2.2. Surface Nanostructuring

The surface structuring of the steel parts was carried out by first making an etch mask using nanosphere lithography and then electrochemically etching the steel. Nanosphere lithography was carried out using conditions previously described [31]. For the targeted structure size, polystyrene particles with diameter of 1 μm and 522 nm were used (Microparticles GmbH, Berlin, Germany). The particles were used as templates for the fabrication of an etch mask suitable for electrochemical etching. A metal oxide layer with a thickness of a few nanometers was deposited on the bead template and a lift-off was carried out, resulting in a nanoporous etch mask. Steel etching was carried out using conventional electrochemical dissolution conditions [26,27].

### 2.3. Hot Embossing and Injection Molding

Hot embossing was used to fabricate nanostructured reference surfaces for wettability and immunoassay trials. Polycarbonate (Makrolon 2207, Bayer, Leverkusen, Germany) was used. Polycarbonate was heated to 10 °C above its glass transition temperature and an embossing pressure of 1.5 MPa was used. The sample was cooled to 20 °C below its glass transition temperature before demolding.

Injection molding was used for the replication of the bio-diagnostic platform, using the material polycarbonate Makrolon 2207 (Bayer). The polymer material was dried at 120 °C for 4 h prior the injection molding. Injection molding was performed using an Engel 50 ton injection molding machine. The nozzle temperature was set at 270 °C. In order to improve the replication quality, a rapid heating and cooling process, based on highly pressurized water, was used. Water temperatures of 60 °C (low) and 170 °C (high) were set, resulting in a mold cavity temperature of 90 °C and 150 °C, respectively. The removal of trapped gas in the mold cavity was performed with a mobile vacuum system, enabling a vacuum level down to 50 mbar in the mold. The average injection pressure was 1600 bars and the cycle time was around 50 s.

### 2.4. Surface Characterization

Structured steel surfaces and polycarbonate replicas were characterized by atomic force microscopy (AFM Dimension Icon, Bruker, CA, USA). Tapping mode AFM was used for the characterization of topography using aluminum-coated silicon tips (typical force constant of 5 N/m) obtained from Budget sensors (Tap150Al-G, Sofia, Bulgaria). The root mean square (RMS) roughness, feature diameter/height/density and image surface area difference were all measured using the built-in functions of the NanoScope software. The image surface area difference corresponds to the difference between the image’s three-dimensional surface area and its projected two-dimensional surface area. This was used to quantify the increase in specific surface area due to the presence of surface features. Scanning electron microscopy (XL-30 ESEM-FEG, Philips, The Netherlands) was used to characterize the structures produced on the 3D steel inserts and the polycarbonate replicas.

### 2.5. Wettability Measurements

The wettability changes of the surfaces were characterized by measuring the contact angle of water sessile droplets deposited on the sample. Advancing and receding water contact angles were determined using a Drop Shape Analysis System DSA10 provided by Krüss (Hamburg, Germany). Standard deviations were calculated using three measurements and the error bars shown on the graphs correspond to the 95% confidence intervals.

### 2.6. Immunoassay

The model immunoassay was carried out by first spotting mouse immunoglobulin (IgG, Jackson ImmunoResearch Europe, Newmarket, UK) using a Nano-PlotterTM (GeSIM, Großerkmannsdorf, Germany). The surface was blocked using bovine serum albumin (BSA) to prevent non-specific adsorption (Jackson ImmunoResearch Europe). The excess BSA was then removed by rinsing with phosphate-buffered saline (PBS) with a surfactant (Tween20) and then PBS only. PBS and PBS-Tween were supplied by Sigma Aldrich (Buchs, Switzerland). The complementary antibody (αIgG, Jackson ImmunoResearch Europe) conjugated to a fluorescent marker (Cy5) was then added. After a last rinsing step (PBS/Tween 20 and PBS) to remove any αIgG excess, a confocal microscope (TCS-SP5, Leica, Heerbrug, Switzerland) was used to image the detection spots. The amount of fluorescence was characterized by measuring the camera-gain necessary to barely reach saturation.

## 3. Results and Discussion

### 3.1. Surface Structuring of Flat Stainless Steel (2D Substrate)

The first part of this study focused on the fabrication of sub-micro-/nanostructures on flat, stainless steel substrates.

**Figure 1:**
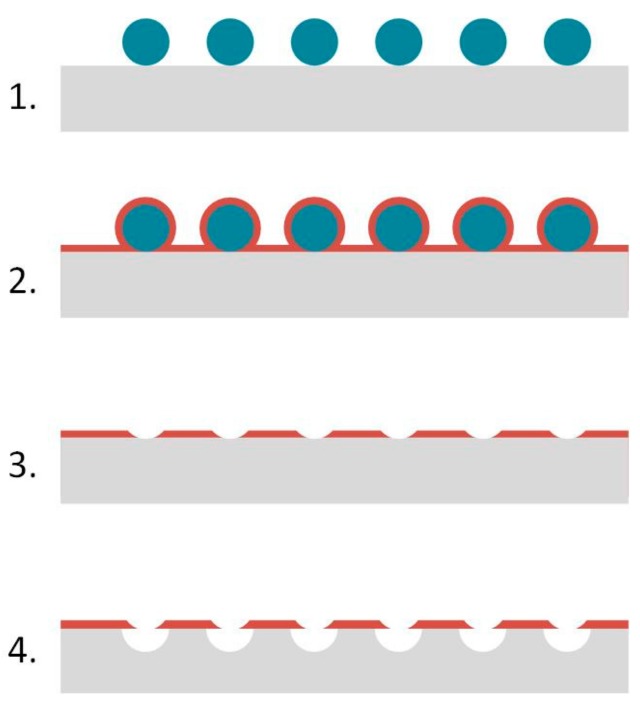
Schematic showing the process flow used for steel nanostructuring.

As shown in Figure 1, the proposed process flow consists of four main steps: first, the deposition of micro-/nanoparticles on the surface of the steel; second, the deposition of a thin (a few nanometers thick) metal oxide layer; third, a lift-off step to remove the particles and produce a porous metal oxide etch mask. Finally, the structures are transferred into the substrate by etching the stainless steel by means of an electrochemical dissolution process. The lateral dimensions are controlled via the particle deposition step (particle diameter, particle density). The vertical dimensions are given by the etching step (depth, profile).

**Figure 2:**
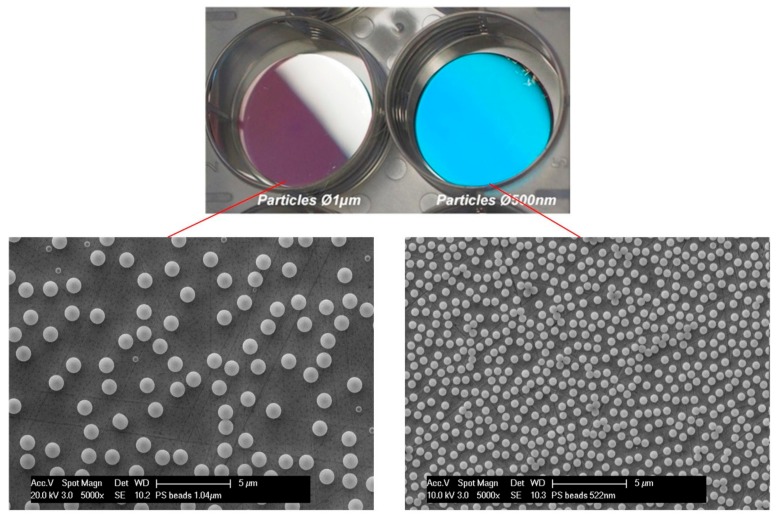
(**Top**) photograph of flat stainless steel inserts coated with 1 μm and 500 nm particles. Bottom (**left**) SEM images of the 1 μm particle monolayer. Bottom (**right**) SEM images of the 500 nm particle monolayer.

**Figure 3:**
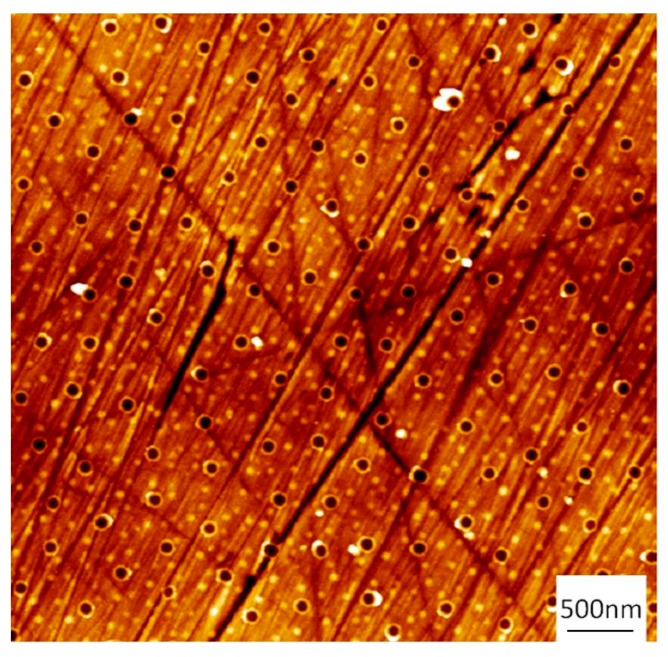
Atomic force microscopy (AFM) image of the stainless steel surface after the deposition of the etch mask.

In Figure 2, photographs and SEM images of flat stainless steel (316L) surfaces coated with particles are presented. The presence of the particles leads to a color change effect due to light scattering. These color changes depend on the size of the particles and the viewing angle and are well described [31]. For 500 nm particles, a monolayer was obtained with a particle density of 1.49 × 10^8^ part·cm^−2^ and a fill factor of 33%. For 1 μm particles, a density of 2 × 10^7^ part·cm^−2^ was obtained with a fill factor of 17%. The particle monolayer was then used for the fabrication of an etch mask before the electrochemical dissolution of the steel. The pattern was transferred into a thin hard mask prior to the electrochemical dissolution. Figure 3 presents the result obtained using 500 nm beads as templates.

**Figure 4:**
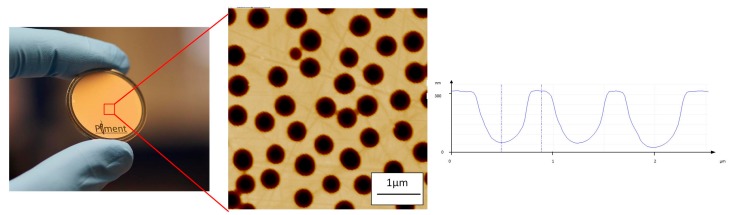
Photograph and AFM images of a flat stainless steel inserts after electrochemical etching.

The samples were then electrochemically etched. In Figure 4, a photograph of the steel surface after etching and the corresponding AFM image of the topography are presented. As for the particle-coated surfaces, the transfer of the structures into the steel gave a color change effect on the surface. AFM confirmed that hemispherical structures were obtained. For an etch mask made using a template of 500 nm beads, the average diameter of the final structures after etching was 460 nm with an average depth of 260 nm. One of the main advantage of electrochemical dissolution is the surface quality obtained in the etched areas, which leads to an RMS roughness on the order of a few nanometers [26]. A range of structures has been fabricated on these flat reference steel substrates using different particles as templates and different etching conditions. Hemispherical structures with diameters of 460 nm and 950 nm have been produced with aspect ratios of up to 1:2.

### 3.2. Surface Nanostructuring of the Microstructured Insert (3D Substrate)

**Figure 5:**
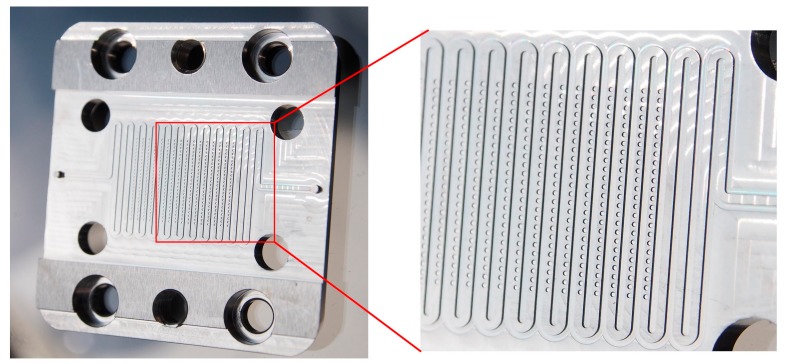
Photograph of the stainless steel insert of the bio-diagnostic platform made by micromilling.

The second part of this study focused on the fabrication of sub-micro-/nanostructures on top of the detection spots of an injection-molded bio-diagnostic platform. To this end, the microstructured steel insert used for injection molding had to be processed. In Figure 5, a photograph of the stainless steel insert fabricated by micromilling is presented. The insert had four mounting-holes for its integration in the mold, holes for the ejector and a micro-ridge corresponding to the microchannel of the bio-diagnostic platform. The array of microholes is clearly visible on top of the ridge. The bottoms of the microholes were electropolished after micromilling to reduce their surface roughness.

**Figure 6:**
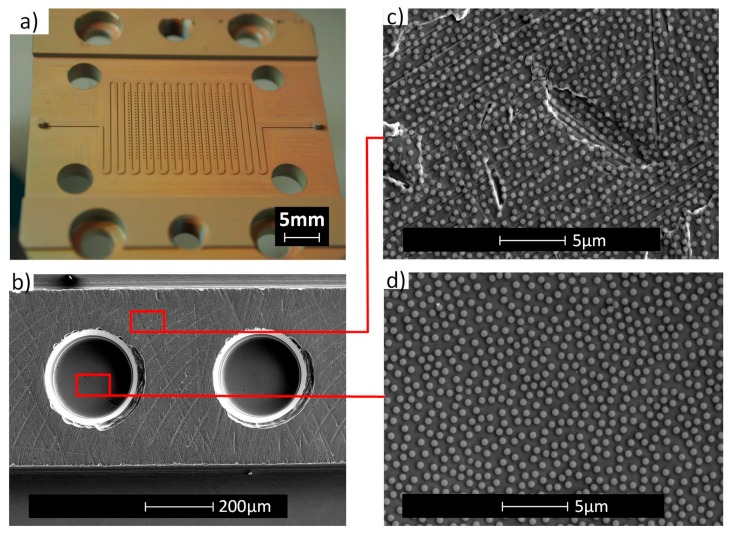
Photograph (**a**) and SEM images (**b**–**d**) of the stainless steel insert of the bio-diagnostic platform coated with particles.

The same process as was used for planar substrates was applied to the insert for the deposition of particles (500 nm diameter). As shown in Figure 6, a homogeneous deposition is obtained on the insert. Particles are observed at the bottom of the holes and on top of the ridge. The background roughness does not influence the particle deposition process and particles can also be observed on the side walls of the microholes (data not shown). The particle density at the bottom was 1.43 × 10^8^ part·cm^−2^ with a fill factor of 25%. This is comparable to the results obtained on a flat surface.

**Figure 7:**
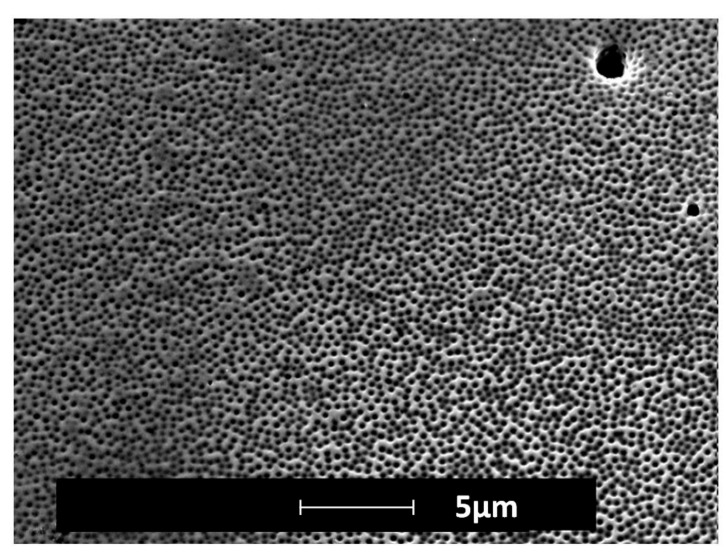
SEM image of the bottom of a microhole of the insert after electrochemical etching.

After the fabrication of the etch mask and electrochemical etching, sub-micro-/nanoholes were observed at the bottom of the microholes (Figure 7). SEM was used to qualitatively examine the structures created at the bottom of the holes. AFM could not be used to make the surface characterization within the microholes. A combination of the high aspect ratio of the holes and the geometry of the AFM cantilever did not allow engagement of the tip at the bottom of the microhole.

### 3.3. Hot Embossing and Injection Molding of Plastic Parts

**Figure 8:**
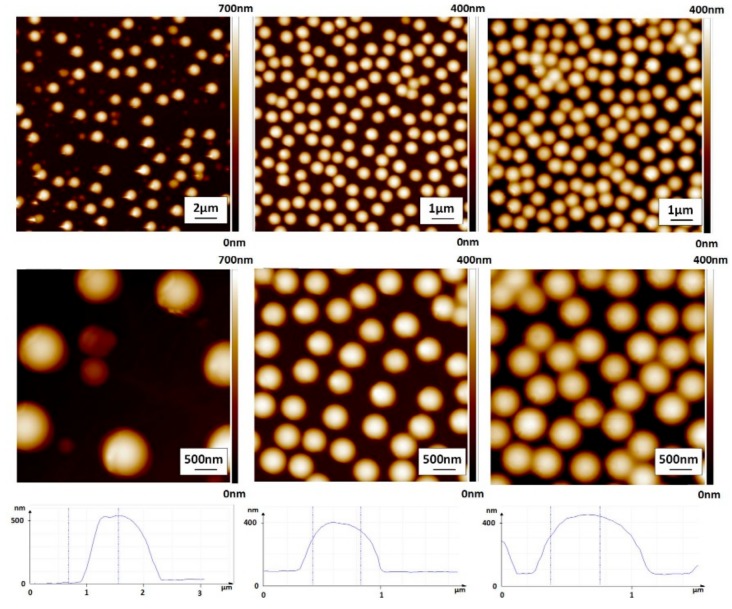
AFM images of the replica hot embossed using three different structured inserts. ((**left**): structure 1; (**middle**): structure 2; (**right**): structure 3.

The structured 2D insert and the bio-diagnostic platform insert were used as molds for replication into a single thermoplast (polycarbonate Makrolon 2207). Hot embossing was used for the nanostructured 2D substrate and injection molding was used for the bio-diagnostic platform. Figure 8 presents the AFM characterization of a hot embossed replica. As expected, sub-micro hemispherical bumps were obtained, the height of which corresponded to the depth of the holes fabricated in the insert. However, the AFM sections show that, while the smallest structures were easily demolded, the largest structures showed side wall defects due to issues with the demolding.

**Figure 9:**
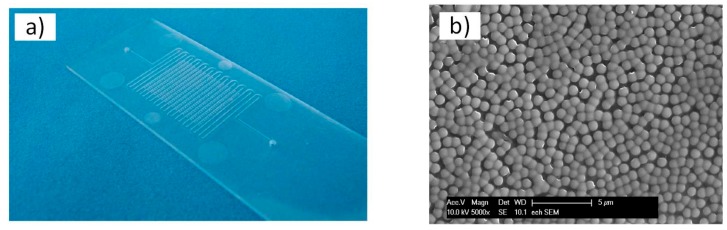
(**a**) Photograph of the injection-molded bio-diagnostic platform. (**b**) SEM image of the nanostructures on the spot of the injection-molded bio-diagnostic platform.

In the case of the bio-diagnostic platform, nanostructures were injection-molded. Figure 9 presents an SEM image of the structures obtained on top of the microdetection spot. The nanostructures were successfully replicated in the polycarbonate.

However, due to characterization limitations, a quantitative assessment of the replication is impossible. The nanostructured surface to be characterized (the top of the microdetection spots) on the polycarbonate replica could not be reached by the AFM tip due to its location within the microchannel of the device.

### 3.4. Characterization

Two types of characterization were carried out. First, the wettability of the samples produced by hot embossing was characterized by measuring water contact angles. Second, a complete immunoassay was performed on all samples to investigate the effect of surface structuring on the sensitivity of the bio-diagnostic platform.

#### 3.4.1. Surface Characterization

Table 1 presents the results of the AFM characterization. The RMS roughness, surface area difference, feature diameter, feature height and feature density have been measured for all three structures from the AFM images. The surface area difference of structure 1 is only 14.7%, whereas it is above 30% for structures 2 and 3. This can be explained by the low feature density of structure 1, which is an order of magnitude lower than that of structures 2 and 3. When we compared the three structures in terms of feature density, we found that structure 1 has the lowest feature density, followed by structures 2 and 3, which have similar values. Concerning the diameter and height of the feature, structure 1 has the largest, followed by structure 3 and then structure 2. These data show that we have three different cases; a low feature density with large structures (structure 1), a high feature density with small structures (structure 2) and finally a high feature density with large structures (structure 3).

#### 3.4.2. Wettability

**Figure 10:**
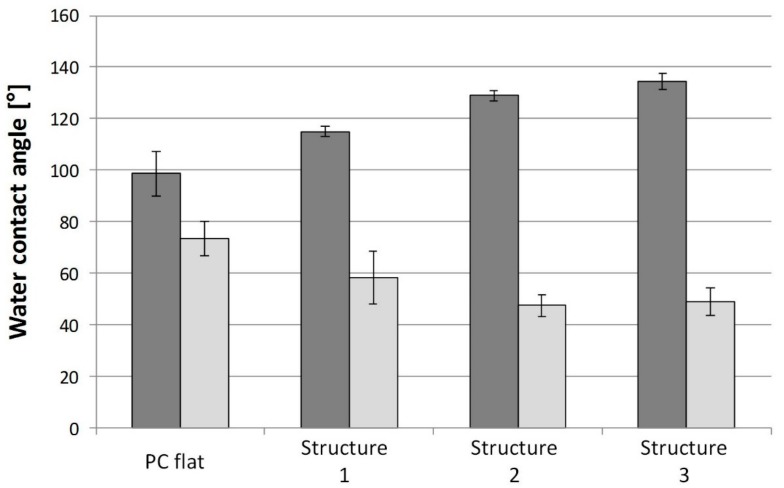
Water contact angles: advancing (dark grey) and receding (light grey) measured on polycarbonate with four different types of structures.

Polycarbonate samples with three different structures were tested. A flat polycarbonate surface was also used as a reference. Wettability is characterized by measuring advancing and receding contact angles of water used as probe-liquid. The results are presented in Figure 10.

For the flat reference, an advancing contact angle of almost 100° was observed with a wetting hysteresis of 30°. The surface micro-/nanostructures lead to not only an increase in the advancing contact angle, but also a significant increase in the contact angle hysteresis for all structures tested. For structure 1, an advancing contact angle of 115° and a hysteresis of 57° were measured. For structures 2 and 3, an advancing contact angle of 135° and a hysteresis of 85° is obtained. This increase in hysteresis suggests that the water drops are in the Wenzel mode (complete wetting of the structure), which corresponds to an increase in the adhesion of the drop on the surface [5]. One objective of the surface structuring is to control the wetting of the solution during the spotting step of the immunoassay. To characterize this, a solution of fluorescently labelled proteins was inkjet printed onto flat and structured polycarbonate surfaces. No significant difference was observed between the flat control and the structured samples.

#### 3.4.3. Immunoassay

**Figure 11:**
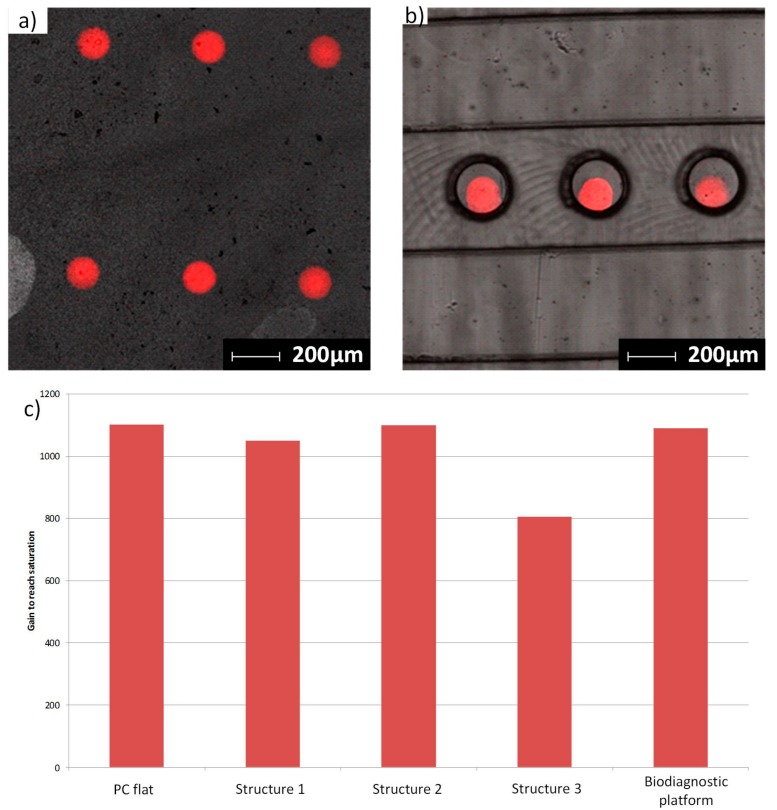
Confocal images of the fluorescent spots printed on a hot embossed sample (**a**) and injection-molded samples (**b**). (**c**) Graph presenting the gains necessary to reach saturation on the fluorescent camera.

The effect of surface micro-/nanostructuring on the sensitivity of a standard immunoassay was investigated. The protocol was applied to either the micro-/nanostructured hot-embossed surfaces or to the detection spots of the injection-molded bio-diagnostic platforms. Fluorescence microscopy was used to characterize the homogeneity of the spots. The sensitivity of the immunoassay was characterized by measuring the gain necessary to reach saturation on the camera. The results are presented in Figure 11. The quantification of the fluorescence signal revealed that the presence of structure 3 caused an increase in the sensitivity of the immunoassay. Compared to the flat reference, the gain necessary to reach saturation was lowered by 30%. The hypothesis initially proposed to explain this effect is that it is due to the increase in the specific surface area resulting from the surface structures. However, the AFM characterization of the structured surfaces revealed 15%, 33% and 37% increases in specific surface area for structures 1, 2 and 3, respectively. Although they have similar specific surface areas, structure 2 and structure 3 did not lead to a similar increase in fluorescence intensity. A second hypothesis is that variation in surface roughness has an effect on the adsorption and conformation of proteins on the surfaces. As shown by Scoppeliti et al., surface roughness can significantly affect the adsorption of proteins during immunoassays and can lead to increased protein density on the surface, which may increase the sensitivity of the immunoassay [32]. A third hypothesis is that the interaction of light with the surface structures may influence the fluorescence emission level. Highly sensitive sensors have been produced using optical interference to improve fluorescence signal [33]. In a previous publication, we showed that particle monolayers deposited on a surface lead to significant optical effects due to the interference between the incident light and the light scattered by the particles [31]. This size-dependent effect is also expected to have occurred with the hemispherical structures of this study. One other hypothesis, therefore, is that the signal enhancement is due to an optical interference effect caused by the presence of the structures. A more detailed analysis of the optical properties of the structured surfaces in the immunoassay medium is needed to confirm this hypothesis and the origin of this enhancement.

## 4. Conclusions

The fabrication of micro-/nanostructured steel surfaces has been achieved by combining nanosphere lithography and electrochemical etching. Structures with lateral sizes of 400 nm to 1 μm with an aspect ratio of 1:2 were produced. The process was applied to planar substrates as well as micromilled inserts presenting micro-ridges and microholes. The material used was stainless steel and it is planned to extend this approach to the structuring of tool-steel used for molds. Polycarbonate replicas were produced by hot embossing or injection molding. The wettability of the surfaces was influenced by the surface structures and an increase in the adhesion of water drops was observed (drops adopted the Wenzel wetting state). One of the structures was also found to significantly increase the sensitivity of an immunoassay, with a 30% increase in fluorescence signal.

## Figures and Tables

**Table 1:** Root mean square (RMS) roughness, surface area difference, average feature diameter and height of the three structures.

ID	RMS Roughness (nm)	Surface Area Difference (%)	Feature Density (part/cm^2^)	Average Feature Diameter (nm)	Average Feature Height (nm)
Structure 1	143	14.7	1.5e7	1249	516
Structure 2	107	33.3	1.37e8	753	287
Structure 3	121	37.3	1.34e8	914	360

## References

[1] Kim S., Jung U.T., Kim S.K., Lee J.H., Choi H.S., Kim C.S., Jeong M.Y. (2015). Nanostructured Multifunctional Surface with Antireflective and Antimicrobial Characteristics. Appl. Mater. Interfaces.

[2] Weder G., Blondiaux N., Giazzon M., Matthey N., Klein M., Pugin P., Heinzelmann H., Liley M. (2010). Use of Force Spectroscopy to Investigate the Adhesion of Living Adherent Cells. Langmuir.

[3] Roch T., Weihnacht V., Scheibe H.J., Roch A., Lasagni A.F. (2013). Direct Laser Interference Patterning of tetrahedral amorphous carbon films for tribological applications. Diam. Relat. Mater..

[4] Quéré D. (2008). Wetting and roughness. Annu. Rev. Mater. Res..

[5] Blondiaux N., Scolan E., Popa A.M., Gavillet J., Pugin R. (2009). Fabrication of superhydrophobic surfaces with controlled topography and chemistry. Appl. Surf. Sci..

[6] Spori D., Drobek T., Zürcher S., Spencer N.D. (2010). Cassie-State wetting investigated by means of holes to pillar density gradient. Langmuir.

[7] Martines E., Seunarine K., Morgan H., Gadegaard N., Wilkinson C.D.W., Riehle M.O. (2005). Superhydrophobicity and superhydrophilicity of regular nanopatterns. Nano Lett..

[8] Blondiaux N., Scolan E., Franc G., Pugin R. (2012). Manufacturing of super-hydrophobic surfaces combining nanosphere lithography with replication techniques. Nanotechnology.

[9] Krishnamoorthy S. (2015). Nanostructured sensors for biomedical applications—A current perspective. Curr. Opin. Biotechnol..

[10] Stewart M.E., Anderton C.R., Thompson L.B., Maria J., Gray S.K., Rogers J.A., Nuzzo R.G. (2008). Nanostructured plasmonic sensors. Chem. Rev..

[11] Ko H., Singamaneni S., Tsukruk V.V. (2008). Nanostructured surfaces and assemblies as SERS media. Small.

[12] Kanipe K.N., Chidester P.P.F., Stucky G.D., Moskovits M. (2016). Large format surface-enhanced Raman spectroscopy substrate optimized for enhancement and uniformity. ACS Nano.

[13] Ingham C.J., Maat J., De Vos W.M. (2012). Where bio meets nano: The many uses for nanoporous aluminum oxide in biotechnology. Biotechnol. Adv..

[14] Kim J.S., Chob J.B., Park B.G., Leed W., Lee K.B., Oh M.K. (2011). Size-controllable quartz nanostructure for signal enhancement of DNA chip. Biosens. Bioelectron..

[15] Kuwabara K., Ogino M., Ando T., Miyauchi A. (2008). Enhancement of fluorescence intensity from an immunoassay chip using high-aspect-ratio nanopillars fabricated by nanoimprinting. Appl. Phys. Lett..

[16] Masuzawa T. (2000). State of the Art of Micromachining. Ann. CIRP.

[17] Bieda M., Schmädicke C., Roch T., Lasagni A. (2015). Ultra-low friction on 100Cr6-steel surfaces after direct laser interference patterning. Adv. Eng. Mater..

[18] Madou M. (2002). Fundamentals of Microfabrication: The Science of Miniaturization.

[19] Pease R.F., Chou S.Y. (2008). Lithography and other patterning techniques for future electronics. Proc. IEEE.

[20] Ruiz R., Kang H., Detcheverry F.A., Dobisz E., Kercher D.S., Albrecht T.R., De Pablo J.J., Nealey P.F. (2008). Density multiplication and improved lithography by directed block copolymer assembly. Science.

[21] Klein M.J.K., Montagne F., Blondiaux N., Vazquez-Mena O., Heinzelmann H., Pugin R., Brugger J., Savu V. (2011). SiN membranes with submicrometer hole arrays patterned by wafer-scale nanosphere lithography. J. Vac. Sci. Technol. B.

[22] Hao L., Meng Y., Chen C. (2014). Experimental investigation on effects of surface texturing on lubrication of initial line contacts. Lubr. Sci..

[23] Mason J.A., Adams D.C., Johnson Z., Smith S., Davis A.W., Wasserman D. (2010). Selective thermal emission from patterned steel. Opt. Express.

[24] Rao P.N., Kunzru D. (2007). Fabrication of microchannels on stainless steel by wet chemical etching. J. Micromech. Microeng..

[25] Landolt D., Chauvy P.F., Zinger O. (2003). Electrochemical micromachining, polishing and surface structuring of metals: Fundamental aspects and new developments. Electrochim. Acta.

[26] Zinger O., Chauvy P.F., Landolt D. (2001). Development of titatnium electrochemical microstructuring towards implant applications. Eur. Cells Mater..

[27] Zinger O., Chauvy P.F., Landolt D. (2003). Scale-resolved electrochemical surface structuring of titanium for biological applications. J. Electrochem. Soc..

[28] Shimizu M., Yamada T., Sasaki K., Takada A., Nomura H., Iguchi F., Yugami H. (2015). Anisotropic multi-step etching for large-area fabrication of surface microstructures on stainless steel to control thermal radiation. Sci. Technol. Adv. Mater..

[29] Al-Azawi1 A., Smistrup K., Kristensen A. (2014). Nanostructuring steel for injection molding tools. J. Micromech. Microeng..

[30] Kurihara K., Saitou Y., Souma N., Makihara S., Kato H., Nakano T. (2015). Fabrication of nano-structure anti-reflective lens using platinum nanoparticles in injection moulding. Mater. Res. Express.

[31] Guillaumée M., Liley M., Pugin R., Stanley R. (2008). Scattering of light by a single layer of randomly packed dielectric microspheres giving color effects in transmission. Opt. Express.

[32] Scopelliti P.E., Borgonovo A., Indrieri M., Indrieri M., Giorgetti L., Bongiorno G., Carbone R., Podesta A., Milani P. (2010). The Effect of Surface Nanometre-Scale Morphology on Protein Adsorption. PLoS ONE.

[33] Yasuda M., Akimoto T. (2012). Highly Sensitive Fluorescence Detection of Avidin/Streptavidin with an Optical Interference Mirror Slide. Anal. Sci..

